# Tachykinin Receptor-Selectivity of the Potential Glioblastoma-Targeted Therapy, DOTA-[Thi^8^,Met(O_2_)^11^]-Substance P

**DOI:** 10.3390/ijms24032134

**Published:** 2023-01-21

**Authors:** Janine Suthiram, Ané Pieters, Zulfiah Mohamed Moosa, Jan Rijn Zeevaart, Mike M. Sathekge, Thomas Ebenhan, Ross C. Anderson, Claire L. Newton

**Affiliations:** 1 Department of Radiochemistry, The South African Nuclear Energy Corporation SOC Ltd. (Necsa), Brits 0240, South Africa; 2 Department of Nuclear Medicine, Faculty of Health Sciences, University of Pretoria, Private Bag X323, Gezina 0031, South Africa; 3 Department of Physiology, Faculty of Health Sciences, University of Pretoria, Private Bag X323, Gezina 0031, South Africa; 4 Centre for Neuroendocrinology, Department of Immunology, Faculty of Health Sciences, University of Pretoria, Private Bag X323, Gezina 0031, South Africa; 5 Nuclear Medicine Research Infrastructure NPC, Level 5 Bridge A, Capital Park, Pretoria 0001, South Africa; 6 Steve Biko Academic Hospital, Private Bag X169, Pretoria 0001, South Africa; 7 Deanery of Biomedical Sciences, University of Edinburgh, Edinburgh EH8 9XD, UK

**Keywords:** neurokinin-1 receptor, neurokinin-2 receptor, neurokinin-3 receptor, mas-related G protein-coupled receptor subtype 2, receptor selectivity, DOTA-[Thi^8^,Met(O_2_)^11^]SP, theranostics, glioblastoma multiforme

## Abstract

Radiopharmaceutical development hinges on the affinity and selectivity of the biological component for the intended target. An analogue of the neuropeptide Substance P (SP), 1,4,7,10-tetraazacyclododecane-1,4,7,10-tetraacetic acid-[Thi^8^,Met(O_2_)^11^]-SP (DOTA-[Thi^8^,Met(O_2_)^11^]SP), in the theranostic pair [^68^Ga]Ga-/ [^213^Bi]Bi-DOTA-[Thi^8^,Met(O_2_)^11^]SP has shown promising clinical results in the treatment of inoperable glioblastoma. As the theranostic targeting component, modifications to SP that affect the selectivity of the resulting analogue for the intended target (neurokinin-1 receptor [NK1R]) could be detrimental to its therapeutic potential. In addition to other closely related tachykinin receptors (neurokinin-2 receptor [NK2R] and neurokinin-3 receptor [NK3R]), SP can activate a mast cell expressed receptor Mas-related G protein-coupled receptor subtype 2 (MRGPRX2), which has been implicated in allergic-type reactions. Therefore, activation of these receptors by SP analogues has severe implications for their therapeutic potential. Here, the receptor selectivity of DOTA-[Thi^8^,Met(O_2_)^11^]SP was examined using inositol phosphate accumulation assay in HEK293-T cells expressing NK1R, NK2R, NK3R or MRGPRX2. DOTA-[Thi^8^,Met(O_2_)^11^]SP had similar efficacy and potency as native SP at NK1R, but displayed greater NK1R selectivity. DOTA-[Thi^8^,Met(O_2_)^11^]SP was unable to elicit significant activation of the other tachykinin receptors nor MRGPRX2 at high concentrations nor did it display antagonistic behaviour at these receptors. DOTA-[Thi^8^,Met(O_2_)^11^]SP, therefore has high potency and selectivity for NK1R, supporting its potential for targeted theranostic use in glioblastoma multiforme and other conditions characterised by NK1R overexpression.

## 1. Introduction

Glioblastoma multiforme is one of the most common and most aggressive forms of central nervous system tumour and, despite therapeutic advances, it remains largely incurable [1,2]. A theranostic strategy involving a radiolabelled derivatised 1,4,7,10-tetraazacyclododecane-1,4,7,10-tetraacetic acid (DOTA)-conjugated derivative of Substance P (SP) (DOTA-[Thi^8^,Met(O_2_)^11^]SP) has recently reported success in the imaging and endotherapy of glioblastoma multiforme [3,4]. SP is a member of the tachykinin family of neuropeptides, of which the other classical members are neurokinin-A (NKA) and neurokinin-B (NKB) [5]. SP, an 11-mer neuropeptide (Arg^1^–Pro^2^–Lys^3^–Pro^4^–Gln^5^–Gln^6^–Phe^7^–Phe^8^–Gly^9^–Leu^10^–Met^11^–NH_2_), is widely distributed in the central and peripheral nervous system of mammalian species [6]. Both neuronal and non-neuronal cells can produce SP [7], and it is associated with many different physiological processes, including gut motility and stressor responses such as vasodilation, inflammation [8], infection control [9] and nociception [10]. The biological activity of SP is principally mediated through interactions with the neurokinin-1 receptor (NK1R) [5]. NK1R, neurokinin-2 receptor (NK2R) and neurokinin-3 receptor (NK3R) form the tachykinin receptor sub-family of the G protein-coupled receptor (GPCR) superfamily of cell surface receptors. Each tachykinin receptor has a distinct pharmacological profile despite a high degree of commonality (40–50% sequence homology [11], with up to 70% homology in their transmembrane domains [12]). Each receptor has a cognate tachykinin peptide with which it preferentially interacts (SP has the highest affinity for NK1R, while NKA and NKB have the highest affinity for NK2R and NK3R, respectively), although they act as full agonists, albeit with differing potencies, at all three tachykinin receptors [11,13]. SP is also a low potency agonist at Mas-related G protein-coupled receptor subtype 2 (MRGPRX2), a GPCR expressed on mast cells [14]. This receptor is reported to be involved in mast cell-mediated inflammatory host responses, including neurogenic and cancer-associated inflammation [15,16] and, upon stimulation by SP, is thought to play a role in stimulating the recruitment of immune cells, contributing to neurogenic pain [16]. Its inappropriate activation has been shown to elicit mast cell-mediated allergic-type reactions and has been implicated in such reactions to many licensed peptidergic drugs [17,18].

Modified analogues of SP have been developed with altered activity, potency, receptor selectivity, in vivo half-life and/or lipophilicity, or have enabled conjugation to moieties for imaging/therapeutic purposes [11,19,20,21]. For example, the use of the chelating agents DOTA or glutaric acid (GA) [3] has allowed for DOTA/GA-SP conjugation to different diagnostic or therapeutically active radiometals [22] for application in nuclear medicine (theranostic) procedures. Theranostics is a concept derived from integrating therapeutics and diagnostics into a single system [23], using a diagnostic to identify/characterise the disease and a second radioactive drug to treat the identified disease. The diagnostic imaging can also be applied to assess follow-up treatment or to stage reoccurrence non-invasively and longitudinally. Cell surface proteins can be utilised as targets for such theranostic moieties. This more personalised therapeutic approach has already demonstrated an improvement in patient care and outcome for several indications including many oncological applications [24,25,26,27,28]. 

DOTA-[Thi^8^,Met(O_2_)^11^]SP is reported to be a metabolically stable analogue of SP with high affinity for tissues expressing neurokinin-1 receptors (NK1Rs) [29]. In glioblastoma multiforme NK1Rs are believed to be highly expressed on the tumour cells and also within the tumour neovasculature, conferring the ability to concurrently target vascular and neoplastic structures [30]. Indeed, the theranostic pair [^68^Ga]Ga-/ [^213^Bi]Bi-DOTA-[Thi^8^,Met(O_2_)^11^]SP has shown promising clinical results in the treatment of inoperable glioblastoma. However, its therapeutic effectiveness is fairly constrained by the short half-life of ^213^Bi. Thus, an attractive alternative, actinium-225 (^225^Ac) radiolabeled DOTA-[Thi^8^,Met(O_2_)^11^]SP, has been proposed featuring the longer half-life of ^225^Ac [31]. Indeed, in vitro studies have indicated a high affinity of [^225^Ac]Ac-DOTA-[Thi^8^,Met(O_2_)^11^]SP for isolated glioblastoma stem cells and cell lines derived from human glioblastoma tumours [32,33]. Although therapeutic efficacy may be affected by aspects such as high heterogeneity of the tumour cells (within a tumour and across malignancy grades), a high level of local recurrence, the infiltrative nature of tumours and the effect of the expression of different isoforms of NK1Rs [30,34], the theranostic use of such SP analogues for the treatment of glioblastomas (as first- or second-line/salvage treatment) remains promising. 

Targeted radiotherapy aims to exploit the specificity of the interaction between the radiolabelled ligand and the cell surface receptor, which should be overexpressed in the target [35], and the specificity of the radiolabelled compound to its target is key to this approach. Therefore, determining receptor selectivity is an important prerequisite to the ongoing in vivo/clinical investigations with this SP analogue. To address this, the tachykinin receptor selectivity of DOTA-[Thi^8^,Met(O_2_)^11^]SP was herein evaluated. 

As it would be unfeasible to examine the activity of the analogue in cells representing all potential off-target sites, a model ‘blank’ cell background was utilised to enable the activity of the analogue at individual receptor subtypes to be clearly established.

## 2. Results and Discussion

**Figure 1:**
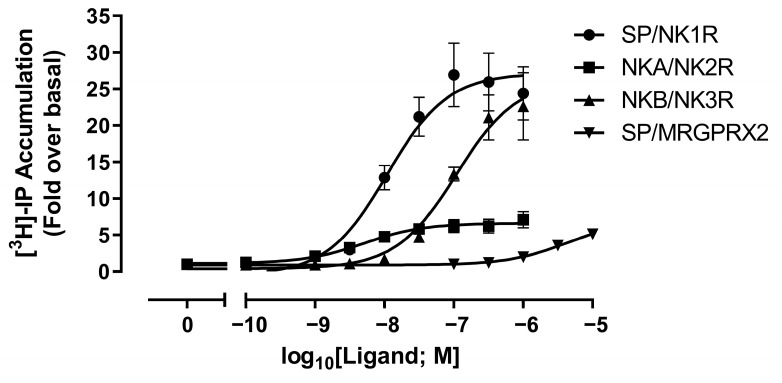
Dose–response analysis of neurokinin-1, -2 and -3 receptor (NK1R, NK2R or NK3R) and Mas-related G protein-coupled receptor subtype 2 (MRGPRX2) activation by cognate ligands. HEK293-T cells transfected with NK1R, NK2R, NK3R or MRGPRX2 were stimulated with a range of concentrations of Substance P (SP): NK1R and MRGPRX2, neurokinin A (NKA): NK2R, or neurokinin B (NKB): NK3R, and receptor activation was measured using an inositol phosphate (IP) accumulation assay. Data are presented as fold-over basal (signal measured in the absence of stimulating ligand; 0) and are presented as mean ± SEM (*n* ≥ 3), in which each data point was performed in triplicate. Data have been fitted to three-parameter sigmoidal dose–response equations used to calculate values for potency (pEC_50_) (Table 1).

Dose-response analyses were conducted to confirm the expression and functionality of the exogenously expressed receptors. NK1R, NK2R, NK3R and MRGPRX2 elicit intracellular signalling responses through coupling to Gα_q/11_ G proteins. Thus, the measurement of inositol phosphate (IP) accumulation (a second messenger generated upon activation of this family of G proteins) was utilised to measure receptor activation in response to ligand stimulation. In cells expressing NK1R or NK3R, a robust and potent (EC_50_: ±10–100 nM) response was elicited by SP or NKB, respectively. A less robust, but potent (EC_50_: ±5 nM) response to NKA was observed in cells expressing NK2R. In cells expressing MRGPRX2, a small response to SP was also observed, but its potency was approximately 4400-fold lower than that observed at the NK1R (EC_50_: ±4 µM) (Figure 1 and Table 1). None of the native ligands elicited similar responses in cells transfected with the empty vector (Supplemental Figure S1), thus the responses observed can be attributed to the presence of the transfected receptors and also serves to confirm the successful exogenous receptor expression in these cells. 

The measurement of receptor expression using ELISA assay confirmed the successful exogenous expression of NK1R, NK2R and NK3R in the HEK293-T cells and indicated that all three are expressed at similar levels (Supplemental Figure S2). Absence of a suitable epitope tag on the MRGPRX2 prohibited similar measurement of this receptor. 

The ability of DOTA-[Thi^8^,Met(O_2_)^11^]SP to activate each of the receptor subtypes was then determined. No response to DOTA-[Thi^8^,Met(O_2_)^11^]SP was observed in cells transfected with the empty vector (Supplemental Figure S1), thus any responses observed can, again, be attributed to the presence of the transfected receptors. 

**Figure 2:**
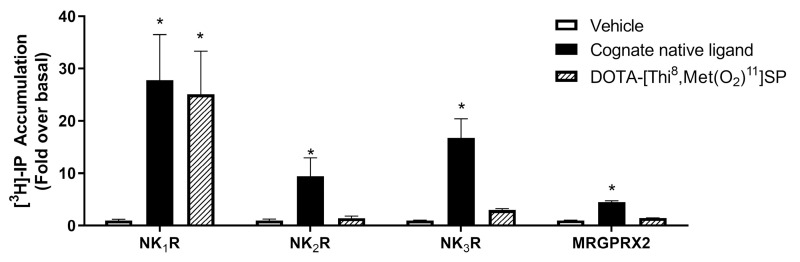
Receptor activation by DOTA-[Thi^8^,Met(O_2_)^11^]SP. HEK293-T cells transfected with NK1R, NK2R, NK3R or MRGPRX2 were incubated with 1 µM (NK1R, NK2R or NK3R) or 10 µM (MRGPRX2) of DOTA-[Thi^8^,Met(O_2_)^11^]SP or native ligand (SP: NK1R and MRGPRX2; NKA: NK2R or NKB: NK3R) and receptor activation was measured using an IP accumulation assay. Data are presented as fold-over-basal (measured in the presence of vehicle) and are mean ± SEM (*n* ≥ 3). * *p* < 0.05 for comparison with vehicle (one-way ANOVA followed by Dunnett’s post hoc test).

**Figure 3:**
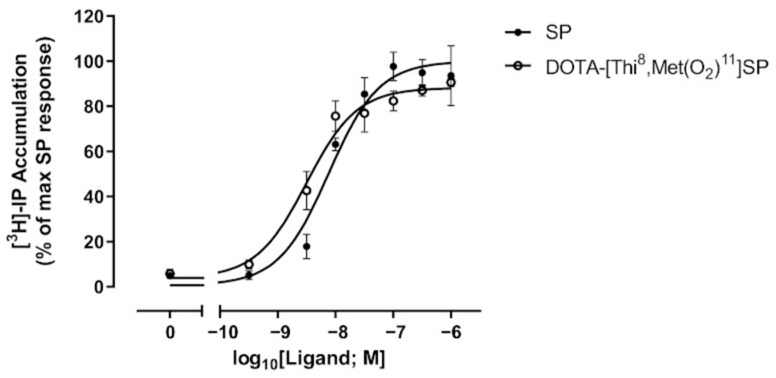
Dose–response analysis of NK1R activation by SP and DOTA-[Thi^8^,Met(O_2_)^11^]SP. HEK293-T cells transfected with NK1R were stimulated with a range of concentrations of SP or DOTA-[Thi^8^,Met(O_2_)^11^]SP and receptor activation was measured using an IP accumulation assay. Data are presented as % of the maximal response elicited by SP and are mean ± SEM (*n* = 3). Data were fitted to three-parameter sigmoidal dose–response equations used to calculate values for potency (pEC_50_) and E_max_ (Table 2).

DOTA-[Thi^8^,Met(O_2_)^11^]SP induced high levels of stimulation of the NK1R, similar to SP at the same concentration (Figure 2) and dose–response analyses revealed that neither the level of stimulation nor the potency of activation induced by DOTA-[Thi^8^,Met(O_2_)^11^]SP was significantly different to that of SP at this receptor (*p* > 0.05; Student’s *t*-test) (Figure 3 and Table 2). These data confirm that the amino acid substitutions in this SP analogue (Phe^8^ to Thi^8^; Met^11^ to Met(O_2_)^11^) together with the N-terminal conjugation of DOTA did not compromise the ligand’s ability to bind effectively to NK1R.

No stimulation by DOTA-[Thi^8^,Met(O_2_)^11^]SP was measured in cells expressing NK2R, NK3R or MRGPRX2 (Figure 2) despite SP being able to elicit a response at these receptors ([11] and Supplemental Figure S3). Although no stimulation of NK2R, NK3R nor MRGPRX2 was observed at the high doses (1–10 µM) utilised in this study, this does not exclude the possibility that activation of these receptors would be seen at even higher concentrations. However, these concentrations would not be clinically relevant and, indeed, much lower concentrations would be utilised for radiopharmaceutical application as in many other theranostic radionuclide therapies [36].

**Figure 4:**
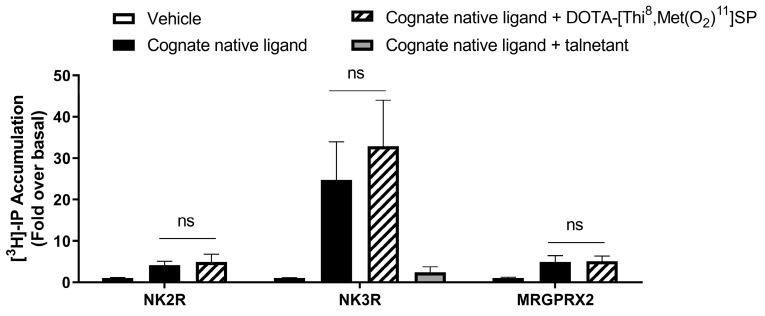
The effect of DOTA-[Thi^8^,Met(O_2_)^11^]SP on activation of NK2R, NK3R and MRGPRX2 by their native ligands. HEK293-T cells transfected with NK1R, NK2R, NK3R or MRGPRX2 were pre-incubated in the presence/absence of 1 µM DOTA-[Thi^8^,Met(O_2_)^11^]SP prior to incubation in the presence/absence of cognate native tachykinin ligand (NK2R: 10 nM NKA, NK3R: 30 nM NKB and MRGPRX2: 10 µM SP) and receptor activation was measured using IP accumulation assay. Data are presented as fold-over basal (measured in the presence of vehicle during pre-incubation and stimulation steps) and are mean ± SEM (*n* = 4). Addition of 1 µM talnetant in place of DOTA-[Thi^8^,Met(O_2_)^11^]SP in cells transfected with NK3R served as a positive control in two experiments (talnetant data are, therefore, mean ± range and were excluded from statistical analysis). ns *p* > 0.05 for comparison with cognate native ligand alone (Student’s *t*-test).

While DOTA-[Thi^8^,Met(O_2_)^11^]SP did not induce activation of NK2R, NK3R, and MRGPRX2, it is possible that this compound is able to bind to these receptors and, thus, may act as an antagonist and inhibit the activity of the native ligands. Antagonist activity of DOTA-[Thi^8^,Met(O_2_)^11^]SP was, therefore, examined. When cells expressing each receptor were stimulated with their cognate native tachykinin ligands in the presence of a high concentration (1 µM) of DOTA-[Thi^8,^Met(O_2_)^11^]SP, no inhibition of receptor activation was observed (Figure 4). Conversely, in the presence of talnetant, a known antagonist of NK3R, NKB-induced NK3R activation was completely abolished, confirming that this methodology was appropriate for the measurement of receptor antagonism.

Although previous studies with DOTA-[Thi^8^, Met(O_2_)^11^]SP have demonstrated activity of this SP analogue on glioblastoma cell lines and stem cells, which have high expression of NK1Rs, to our knowledge, no investigation into the receptor specificity of this modified SP analogue has been undertaken. Modification of the tachykinin peptides can significantly alter their receptor selectivity profiles [37] and, thus, can have an adverse impact on the target-to-non-target ratio of therapeutic/diagnostic agents. Structure–activity relationship studies have revealed that the common C-terminal motif of the tachykinins (Phe–X–Gly–Leu–Met-NH_2_, where X is an aromatic [Phe/Tyr] or branched chain aliphatic [Val/Ile] residue), is responsible for tachykinin receptor binding/activation, while their distinct N-terminal portions determine receptor subtype selectivity [11,21]. With respect to the SP analogue examined herein (DOTA-[Thi^8^, Met(O_2_)^11^]SP), the amino acids in positions 8 and 11 (which correspond to the X position and terminal Met, respectively) of the common tachykinin motif have been substituted. Thus, it may be hypothesised that these substitutions could affect receptor interactions generally rather than subtype selectivity. However, this was not the case as the potency of this analogue at the NK1R was unaffected. Indeed, as confirmed here, oxidation of the sulphur of Met^11^ of SP to sulfone (Met(O_2_)^11^) has previously been demonstrated to increase selectivity for the NK1R [20]. This analogue also has been demonstrated to have increased lipophilicity and in vivo half-life (a desirable feature of a biomarker for radiopharmaceutical application) compared to native SP [19]. 

In conclusion, the data presented herein confirm that DOTA-[Thi^8^,Met(O_2_)^11^]SP is an NK1R agonist and that it displays increased selectivity for this receptor subtype compared to the cognate native ligand, SP, which should translate to a higher target-to-non-target ratio, supporting its continued therapeutic development for use in NK1R-targeted theranostic approaches for glioblastoma multiforme. Furthermore, as many different tumour types have been shown to have increased NK1R expression [38,39,40,41,42,43,44], it is possible that such theranostic approaches could be applied to other cancer types, or, indeed, to other conditions characterised by NK1R overexpression. We have recently described a simple kit-like formulation of DOTA-[Thi^8^, Met(O_2_)^11^]SP [45], as a further step towards a user-friendly tracer preparation, enabling efficient clinical investigations of radiolabelled DOTA-[Thi^8^, Met(O_2_)^11^]SP analogues in the future. Although the primary tachykinin receptor signal transduction mechanism (PLC activation) was utilised to measure receptor activation/inhibition in the present study, future pre-clinical studies further examining the pharmacology of this SP analogue could explore differential/biased signalling using different signalling modalities elicited by activation of these receptors [46] and/or the activation of different NK1R isoforms [34].

## 3. Materials and Methods

### 3.1. Materials

pcDNA mammalian expression vectors encoding human NK3R (GenBank accession number: AY462098), NK2R (GenBank accession number: AY322545), NK1R (GenBank accession number: AY462099) and MRGPRX2 (GenBank accession number: NM_054030) were obtained from the Bloomsburg University cDNA Resource Center (www.cdna.org [accessed on 18 October 2018]). The NK1R, NK2R and NK3R vectors included an N-terminal 3xHA epitope tag. The empty vector, pcDNA3.1, was obtained from Thermo Fisher Scientific (Waltham, MA, USA). Peptide ligands, NKA (H-His-Lys-Thr-Asp-Ser-Phe-Val-Gly-Leu-Met-NH_2_ (trifluoroacetate salt), 1133.34 g/mol) and NKB (H-Asp-Met-His-Asp-Phe-Phe-Val-Gly-Leu-Met-NH_2_ (trifluoroacetate salt), 1210.44 g/mol) were purchased from CPC Scientific (Sunnyvale, CA, USA), SP (Arg-Pro-Lys-Pro-Gln-Gln-Phe-Phe-Gly-Leu-Met-NH_2_ (acetate salt hydrate), 1347.63 g/mol) from Sigma-Aldrich (St. Louis, MO, USA) and DOTA-[Thi^8^,Met(O_2_)^11^]-SP (DOTA-Arg-Pro-Lys-Pro-Gln-Gln-Phe-Thi-Gly-Leu-Met(O_2_)-NH_2_, 1772.06 g/mol) from piCHEM (Raaba-Grambach, Austria). Talnetant was kindly supplied by Dr Mike Trower (NeRRe Therapeutics, Stevenage, UK). 

### 3.2. Cell Culture

Modified human embryonic kidney cells (HEK293-T; American Tissue and Cell Culture Collection), which provide a ‘blank’ background (with little/no endogenous tachykinin receptor nor MRGPRX2 expression [47]) and stably express large T-antigen for optimal expression of exogenous proteins, were utilised. Cells were cultured in a humidified atmosphere at 37 °C with 5% CO_2_ in Dulbecco’s Modified Eagle Medium containing GlutaMAX™ (DMEM) (Life Technologies, Carlsbad, CA, USA) supplemented with 10% foetal calf serum (FCS) (Life Technologies, Carlsbad, CA, USA). Cells were passaged regularly to maintain them at less than 80% confluence. 

### 3.3. Measurement of Receptor Signalling

The tachykinin receptors elicit intracellular signalling responses primarily through coupling to, and the activation of, Gα_q/11_ G proteins. The activation of these G proteins results in the generation of inositol phosphates (IPs; inositol trisphospate and its metabolites inositol bisphosphate and inositol monophosphate). Loading of cells with a radiolabelled precursor ([^3^H]-myo-inositol) ensures that the generated IPs are radiolabelled. These can be isolated using ion exchange chromatography and their radioactivity measured, with the amount of radiolabelled IP generated (i.e., the level of radioactivity measured) being proportional to the signalling activity of the receptor [48].

#### 3.3.1. Transient Transfection

Cells were seeded at a density of 1.5 × 10^5^ cells/well in 24-well tissue culture plates coated with Matrigel, Growth Factor Reduced Matrix (BD Biosciences, Franklin Lakes, NJ, USA) at a 1:30 dilution in DMEM (to aid cell attachment). To facilitate expression of exogenous receptor proteins, 24 h post-seeding, cells were transfected with plasmids encoding receptors or with empty vector (0.5 µg/well) using X-treme GENE HP (XTG) transfection reagent (Sigma-Aldrich, St. Louis, MO, USA). Transfection complexes were prepared by mixing DNA with XTG at a 1:2 ratio (μg DNA:μL XTG) in DMEM. Following incubation for 15 min at room temperature, the complexes were then added to the cells. 

#### 3.3.2. Inositol Phosphate Accumulation Assay

At 12 h post-transfection, the culture media were replaced with reduced inositol Media 99 (Thermo Fisher Scientific, Waltham, MA, USA), supplemented with 1% FCS and 0.5 μCi/well [^3^H]myo-inositol (PerkinElmer, Waltham, MA, USA). Cells were then incubated for a further 12 h at 37 °C before replacing the media with Buffer I (DMEM supplemented with 20 mM HEPES, 10 mM LiCl (to inhibit IP breakdown) and 0.1% bovine serum albumin) and incubating for 30 min at 37 °C. Media were then aspirated, and cells were incubated with ligands prepared in Buffer I. 

For the measurement of receptor activation, cells were incubated for 1 h at 37 °C in the presence of vehicle (0.1% DMSO), a range of concentrations of DOTA-[Thi^8^,Met(O_2_)^11^]SP or native ligands for dose–response analyses. For single-concentration analyses, cells were incubated with 1 µM [NK1R, NK2R, NK3R and empty vector) or 10 µM [MRGPRX2 and empty vector] DOTA-[Thi^8^, Met(O_2_)^11^]-SP or native ligands. For the measurement of receptor antagonism, cells were incubated for 30 min at 37 °C in the presence of vehicle (0.1% DMSO), 1 µM DOTA-[Thi^8^,Met(O_2_)^11^]SP or 1 µM talnetant ((S)-N-(1-phenylpropyl)-3-hydroxy-2-phenylquinoline-4-carboxamide, also known as SB-223412; a non-peptide antagonist of the NK3R [49]) prior to stimulation for 1 h at 37 °C with native ligands at close to their EC_50_ concentration (Table 1; MRGPRX2, 10 µM SP; NK2R, 10 nM NKA and NK3R, 30 nM NKB). 

Following ligand incubation, cells were lysed using incubation in 10 mM formic acid for 60 min at 4 °C. Lysates were transferred to tubes containing 100–200 mesh Dowex 1X8 resin (Sigma-Aldrich, St. Louis, MO, USA) and incubated for 5 min. The resin was washed twice with water and twice with wash buffer (60 mM ammonium formate, 5 mM sodium tetraborate) before elution of the radiolabelled inositol phosphates from the beads in 1 mL elution buffer (1 M ammonium formate/0.1 M formic acid). A total of 1.5 mL scintillation fluid (OptiPhase HiSafe3) (PerkinElmer, Waltham, MA, USA) was then added and radioactivity (decays per minute; dpm) was determined with liquid scintillation counting using a Packard Tri-Carb 4810TR Liquid Scintillation Analyser (PerkinElmer, Waltham, MA, USA).

#### 3.3.3. Data Analysis

Data were analysed using GraphPad Prism Version 8 (GraphPad, Inc., San Diego, CA, USA). For the dose–response analyses, three-parameter sigmoidal dose–response curves were generated and used to calculate the potency (pEC_50_) and maximal response (E_max_), where appropriate. Statistical comparisons were made using one-way ANOVA or Student’s t-test, as appropriate, with *p* < 0.05 considered significant.

## Figures and Tables

**Table 1:** Dose–response parameters for stimulation of cells expressing NK1R, NK2R, NK3R and MRGPRX2 with cognate native tachykinin ligands.

Ligand	Receptor	pEC_50_ (EC_50_)
SP	NK1R	8.00 ± 0.07 (9.91 nM)
NKA	NK2R	8.30 ± 0.07 (4.99 nM)
NKB	NK3R	6.99 ± 0.10 (102 nM)
SP	MRGPRX2	5.38 ± 0.15 (4170 nM) ^†^

Data are presented as mean ± SEM (*n ≥* 3). SP: substance P; NKA: neurokinin A; NKB: neurokinin B; NK(1/2/3)R: neurokinin-1/-2/-3 receptor; MRGPRX2: Mas-related G protein-coupled receptor subtype 2. ^†^ plateau not achieved so pEC_50_ may not be accurate.

**Table 2:** Dose–response parameters for stimulation of cells expressing NK1R with SP and DOTA-[Thi^8^,Met(O_2_)^11^]SP.

Ligand	Emax	pEC50 (EC50)
SP	100 ± 8	8.13 ± 0.07 (7.41 nM)
DOTA-[Thi8,Met(O2)11]SP	89 ± 1	8.48 ± 0.17 (3.31 nM)

Data are presented as mean ± SEM (*n* = 3). SP: substance P; NK1R: neurokinin-1 receptor. *p* > 0.05 (not significant) for comparison with SP.

## Data Availability

The data presented in this study are available on request from the corresponding author (C.L.N.).

## Supplementary Materials

The supporting information can be downloaded at: https://www.mdpi.com/article/10.3390/ijms24032134/s1.
