# Supplementary material for: Tachykinin Receptor-Selectivity of the Potential Glioblastoma-Targeted Therapy, DOTA-[Thi8,Met(O2)11]-Substance P
Source: Int J Mol Sci. 2023 Jan 21;24(3):2134. doi: 10.3390/ijms24032134 (PMC9916806; doi:10.3390/ijms24032134)
Supplement: Supplementary file 1 [file ijms-24-02134-s001.zip › ijms-2058104-supplementary.pdf]

Supplementary Data

The stimulation of cells transfected with empty vector by NKA, NKB, SP and DOTA-[Thi^8,Met(O$_2$)^{11}]SP was measured by IP accumulation assay as described in Section 3.3.

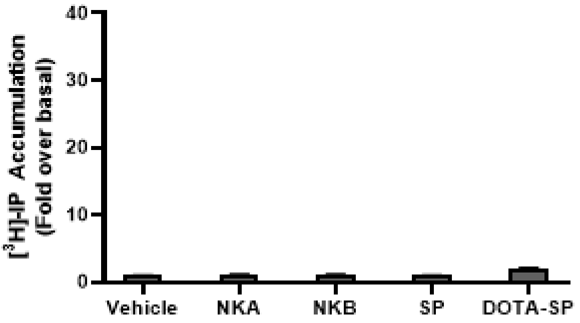

Supplemental Figure S1. Activation of empty-vector-transfected cells with NKA, NKB, SP and DOTA-[Thi^8,Met(O$_2$)^{11}]SP. HEK293-T cells transfected with empty vector were incubated with 1 μM (NKA, NKB or SP) or 10 μM (DOTA-[Thi^8,Met(O$_2$)^{11}]SP (DOTA-SP)). Activation was measured using an IP accumulation assay. Data are presented as fold-over-basal (measured in the presence of vehicle) and are mean ± SEM ($n \geq 3$).

A receptor enzyme-linked immunosorbent assay (ELISA) was utilised to measure receptor expression at the cell surface of intact cells (their functional ligand-accessible location). A primary antibody (mouse anti-HA) was used to label the N-terminal epitope tags attached to the extracellular portion of the exogenously expressed NK1R, NK2R and NK3Rs. Absence of a suitable epitope tag on the MRGPRX2 prohibited similar measurement of this receptor.

Cells were seeded at a density of 0.8x10^5 cells/well in 48-well tissue culture plates coated with Matrigel, Growth Factor Reduced Matrix (BD Biosciences, Franklin Lakes, NJ, USA) at a 1:30 dilution in DMEM (Life Technologies, Carlsbad, CA, USA) (to aid cell attachment). To facilitate expression of exogenous receptor proteins, 24 h post-seeding, cells were transfected with plasmids encoding receptors or with empty vector (0.3 μg/well) using X-treme GENE HP (XTG) transfection reagent (Sigma-Aldrich, St. Louis, MO, USA). Transfection complexes were prepared by mixing DNA with XTG at a 1:2 ratio (μg DNA:μl XTG) in DMEM. Following incubation for 15 minutes at room temperature, the complexes were then added to cells and incubated for 24 hours (to allow for adequate receptor expression).

24 h post-transfection, cells were washed with phosphate buffered saline (PBS; 137 mM NaCl, 2.7 mM KCl, 10 mM Na_2HPO_4, 1.8 mM KH_2PO_4, pH 7.4) supplemented with 0.5 mM $MgCl_2$ and 0.9 mM $CaCl_2$ before fixation by incubation with 2% (w/v) paraformaldehyde at room temperature for 15 min. Cells were then washed thoroughly with PBS (3x 10 minute washes) before incubation with blocking solution (DMEM supplemented with 10% FCS (v/v) (Life Technologies, Carlsbad, CA, USA), 5% (w/v) bovine serum albumin and 5% (w/v) skim milk powder) for 1 hour at 37°C. Cells were then washed thoroughly prior to incubation with primary antibody (mouse anti-HA, Life Technologies, Carlsbad, CA, USA [#32-6700], diluted 1:1000 in DMEM supplemented with 10% FCS) for 16 h at 4°C. Following a further thorough washing with PBS, cells were incubated with secondary antibody (goat anti-mouse conjugated to horseradish peroxidase, Bio-Rad Laboratories, Hercules, CA, USA [#1706516], diluted 1:2000 in DMEM supplemented with 10% FCS) for 1 h at 37°C. Cells were then washed again thoroughly with PBS before incubation for 10 min in the dark with 0.1 mg/ml 3,3′,5,5′-tetramethylbenzidine in phosphate citrate buffer (50 mM citric acid, 102 mM Na_2HPO_4, pH 5.0), supplemented with 0.006% (v/v) H_2O_2. Sulfuric acid (1M final concentration) was added to stop the reactions and absorbance was measured at 450 nm using an iMark microplate reader (Bio-Rad Laboratories, Hercules, CA, USA). Data were analysed using GraphPad Prism Version 8 (GraphPad,

Inc., San Diego, CA, USA) and were calculated as fold-over background (measured in cells transfected with empty vector only).

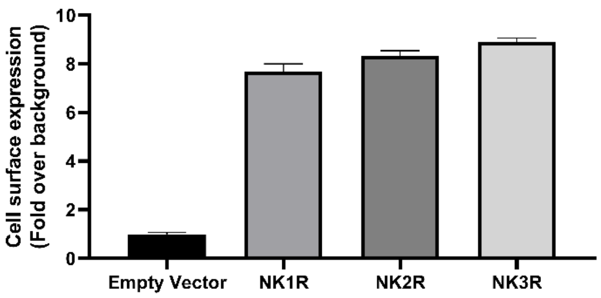

Supplemental Figure S2. Receptor cell surface expression. Receptor cell surface expression in HEK293-T cells transfected with NK1R, NK2R or NK3R was determined by receptor ELISA assay utilising anti-HA primary antibodies and HRP-conjugated secondary antibodies. Data are presented as fold-over background (measured in cells transfected with empty vector only) and are mean ± SEM (n=6).

The stimulation of cells transfected with NK2R and NK3R by SP and DOTA-[Thi^{8},Met(O$_2$)^{11}]SP was measured by IP accumulation assay as described in Section 3.3.

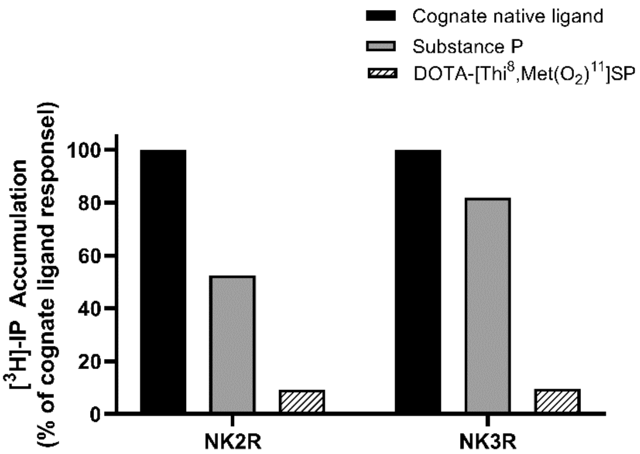

Supplemental Figure S3. Activation of NK2R or NK3R transfected cells with SP and DOTA-[Thi^{8},Met(O$_2$)^{11}]SP. HEK293-T cells transfected with NK2R or NK3R were incubated with 1 μM cognate native ligand (NKA: NK2R or NKB: NK3R), SP or DOTA-[Thi^{8},Met(O$_2$)^{11}]SP. Activation was measured using an IP accumulation assay. Data are presented as a percentage of the cognate native ligand response and are from a single experiment (*n*=1).
